# *Aspergillus*-positive lower respiratory tract samples in patients with the acute respiratory distress syndrome: a 10-year retrospective study

**DOI:** 10.1186/s13613-016-0156-2

**Published:** 2016-06-13

**Authors:** Damien Contou, Matthieu Dorison, Jérémy Rosman, Frédéric Schlemmer, Aude Gibelin, Françoise Foulet, Françoise Botterel, Guillaume Carteaux, Keyvan Razazi, Christian Brun-Buisson, Armand Mekontso Dessap, Nicolas de Prost

**Affiliations:** Groupe Henri Mondor-Albert Chenevier, Centre Hospitalier Universitaire Henri Mondor, DHU A-TVB, Service de Réanimation Médicale, Assistance Publique-Hôpitaux de Paris, 51, Avenue du Maréchal de Lattre de Tassigny, 94010 Créteil Cedex, France; Groupe de Recherche CARMAS, Faculté de Médecine de Créteil, Université Paris Est Créteil, 94010 Créteil, France; Centre Hospitalier Universitaire Henri Mondor, DHU A-TVB, Antenne de Pneumologie, Assistance Publique-Hôpitaux de Paris, 94010 Créteil, France; Unité de Mycologie, Département de Biologie-Pathologie, Centre Hospitalier Universitaire Henri Mondor, DHU VIC, Assistance Publique-Hôpitaux de Paris, 94010 Créteil, France

**Keywords:** *Aspergillus*, Invasive pulmonary aspergillosis, Acute respiratory distress syndrome, Immunosuppression

## Abstract

**Background:**

The detection of *Aspergillus* spp. in endotracheal aspirate cultures of mechanically ventilated patients may reflect either colonization or infection. However, little is known about the prevalence and the impact on outcome of respiratory tract sample positive for *Aspergillus* during the acute respiratory distress syndrome (ARDS).

**Methods:**

We conducted a monocentric, retrospective study over a 10-year period (January 2006–December 2015) in the ICU of a university hospital. All consecutive adult patients with ARDS were included, and the diagnosis of invasive pulmonary aspergillosis was assessed using a previously validated algorithm.

**Results:**

In total, 423 ARDS patients were included with 35 patients [8.3 %, 95 % CI (5.4–10.6)] having at least one respiratory tract sample positive for *Aspergillus* (Aspergillus^+^ patients) after a median delay of 3 days (1–11) following ICU admission. Comorbidities did not differ between Aspergillus^+^ and Aspergillus^−^ patients except for more frequent immunosuppression in Aspergillus^+^ patients (40 vs. 22 %; *p* = 0.02). There was no difference between Aspergillus^−^ and Aspergillus^+^ patients regarding in-ICU mortality, ventilator-free days at day 28, and incidence of ventilator-associated pneumonia, but need for renal replacement therapy was higher in Aspergillus^+^ patients than in others (49 vs. 27 %; *p* = 0.01). Seventeen [4.0 %, 95 % CI (2.1–5.9)] patients had putative/proven aspergillosis. After adjusting on covariates associated with ICU mortality, putative/proven aspergillosis was associated with in-ICU mortality [aOR = 9.58 (1.97–46.52); *p* = 0.005], while *Aspergillus* colonization was not [aOR = 0.64 (0.21–1.99); *p* = 0.44].

**Conclusions:**

Eight percent of ARDS patients had *Aspergillus* spp.-positive respiratory tract cultures. These had a higher risk of mortality only when categorized as having putative or proven invasive pulmonary aspergillosis.

## Background

Invasive pulmonary aspergillosis (IPA) has been reported chiefly in immunocompromised patients with prolonged neutropenia, organ and allogeneic stem cell transplantation, prolonged corticosteroids use or severe inherited immunodeficiency [1]. However, in the past decade, definite cases of IPA have also been reported in intensive care unit (ICU) patients having none of the previously defined host risk factors for IPA [2], but other associated illnesses including advanced cirrhosis [3, 4], H1N1 *Influenza* infection [5] or chronic obstructive pulmonary disease (COPD) [4–7]. In one study, the prevalence of IPA reached 6 % in a cohort of patients without malignancy hospitalized in a medical ICU [4]. Endotracheal aspirate cultures growing *Aspergillus* spp. have been recorded in 1–2 % of mechanically ventilated ICU patients having no predisposing factors and may reflect either colonization or infection [8–11]. A recent clinical algorithm developed by Blot et al. demonstrated favorable operating characteristics to discriminate *Aspergillus* respiratory tract colonization from IPA in ICU patients, whereas the European Organization for Research and Treatment of Cancer/Mycosis Study Group (EORTC/MSG) criteria failed to adequately categorize patients in the absence of conventional risk factors [1].

The acute respiratory distress syndrome (ARDS) [12] occurs in about 10 % of ICU patients and is associated with a high mortality of 35 % [13]. Respiratory tract *Aspergillus* colonization was shown to be more frequent in ARDS than in other critically ill patients [14]. An autopsy study of 64 patients with ARDS revealed that 8 of them (13 %) had died with pulmonary lesions of IPA [15]. However, the burden of IPA during ARDS has been poorly studied and little is known on the prevalence of *Aspergillus* respiratory tract colonization and IPA during ARDS, as well as on the prognosis of IPA in this setting. In this monocenter retrospective study we aimed at: (1) assessing the prevalence, (2) reporting the clinical characteristics, and (3) evaluating the impact on outcome of *Aspergillus*-positive lower respiratory tract specimen in ARDS patients.

## Methods

We conducted a monocenter retrospective study in the 24-bed medical ICU of a tertiary referral center (Henri Mondor Hospital, Créteil, France). All consecutive adult (>18 years) patients admitted in the ICU for ARDS according to the Berlin definition criteria (within 48 h of admission) and receiving invasive mechanical ventilation over a 10-year period (January 2006 to December 2015) were included [12]. Exclusion criteria were as follows: previously known lung interstitial disease or tumoral infiltration, chronic respiratory failure requiring long-term oxygen therapy, pure cardiogenic pulmonary edema, mild ARDS treated with noninvasive ventilation only, proven or suspected invasive pulmonary aspergillosis under antifungal therapy upon ARDS diagnosis and patients for whom no endobronchial sampling had been obtained.

All respiratory tract samples (plugged telescoping catheter, tracheal aspirate or bronchoalveolar fluid) performed for microbiological examination were analyzed. Galactomannan antigen (GM) detection in plasma and in bronchoalveolar lavage (BAL) fluid was performed at the discretion of the managing physician. An optical density ratio of 0.5 or greater for GM in serum and of 1.0 or greater for BAL fluid was considered positive. Chest CT scan and cerebral or facial scan were not routinely performed.

### Definition of infection and categorization of patients

Patients were categorized into two groups: those with one or more respiratory tract sample positive in culture for *Aspergillus spp*. (Aspergillus^+^ patients) during the ICU stay and those without such positive sample (Aspergillus^−^ patients). The former group was further split into three categories depending on the probability of IPA according to the clinical algorithm proposed by Blot et al. [16]: (A) proven IPA (microscopic analysis on sterile material: histopathologic, cytopathologic or direct microscopic examination of a specimen obtained by needle aspiration or sterile biopsy in which hyphae are seen accompanied by evidence of associated tissue damage; isolation of *Aspergillus* from culture of a specimen obtained by lung biopsy); (B) putative IPA in case of (1) *Aspergillus*-positive lower respiratory tract specimen culture (entry criterion) with (2) compatible signs and symptoms (one of the following: fever refractory to at least 3 days of appropriate antibiotic therapy, recrudescent fever after a period of defervescence of at least 48 h while still on antibiotics and without other apparent cause, pleuritic chest pain, pleuritic rub, dyspnea, hemoptysis, worsening respiratory insufficiency in spite of appropriate antibiotic therapy and ventilatory support) and (3) abnormal medical imaging by portable chest X-ray or CT scan of the lungs, and either (4a) a host risk factor (one of the following conditions: neutropenia (absolute neutrophil count <500 G/L) preceding or at the time of ICU admission, underlying hematological or oncological malignancy treated with cytotoxic agents, glucocorticoid treatment (prednisone equivalent >20 mg/day), congenital or acquired immunodeficiency) or (4b) a semiquantitative *Aspergillus*-positive culture of BAL fluid (+ or +++), without bacterial growth together with a positive cytological smear showing branching hyphae or (C) *Aspergillus* respiratory tract colonization when ≥1 criterion necessary for a diagnosis of putative IPA was not met (Tables 1, 2).

**Table 1:** Demographics and clinical characteristics upon ICU admission of ARDS patients with (*Aspergillus* ^+^) or without (*Aspergillus* ^−^) one or more respiratory tract sample positive for *Aspergillus* spp.

	All (*n* = 423)	*Aspergillus* ^−^ (*n* = 388)	*Aspergillus* ^+^ (*n* = 35)	*p* value
Age (years)	62 (50–72)	62 (50–72)	62 (49–72)	0.82
Gender (male)	282 (67)	258 (66)	24 (69)	0.85
Previously known aspergillosis	8 (2)	7 (2)	1 (3)	0.50
Immunosuppression	100 (24)	86 (22)	14 (40)	0.023
COPD	48 (11)	44 (11)	4 (11)	>0.99
Inhaled steroids	16 (4)	15 (4)	1 (3)	>0.99
Liver cirrhosis	44 (10)	42 (11)	2 (6)	0.56
Chronic renal failure	16 (4)	15 (4)	1 (3)	>0.99
Diabetes mellitus	85 (20)	79 (20)	6 (17)	0.83
SAPS II	53 (37–69)	53 (38–70)	51 (34–68)	0.65
LODS	8 (6–12)	8 (6–12)	7 (5–11)	0.26
Main ARDS risk factors				
Pulmonary infection	202 (48)	173 (45)	29 (83)	<0.0001
Aspiration	154 (36)	147 (38)	7 (20)	0.043
Non-pulmonary sepsis	87 (21)	84 (22)	3 (9)	0.080
Drug overdose	12 (3)	11 (3)	1 (3)	>0.99
Delay first respiratory symptom—admission, days	2 (0–5)	2 (0–5)	2 (0–8)	0.41
Temperature > 38.3 °C	220 (52)	199 (51)	21 (60)	0.38
Noninvasive ventilation	69 (16)	65 (17)	4 (11)	0.63
Berlin classification				0.38
Mild	113 (27)	104 (27)	9 (26)	
Moderate	162 (38)	144 (37)	18 (51)	
Severe	148 (35)	140 (36)	8 (23)	
PaO_2_/FiO_2_ ratio (mm Hg)	106 (77–163)	106 (78–162)	114 (76–173)	0.84
Shock	325 (77)	297 (76)	28 (80)	0.83
Serum creatinine (µmol/L)	120 (82–180)	120 (82–177)	128 (82–207)	0.76

*ARDS* acute respiratory distress syndrome, *COPD* chronic obstructive pulmonary disease; continuous variables are shown as median (interquartile range 25–75); categorical variables are shown as *n* (%)

**Table 2:** Classification of ARDS patients with one or more respiratory tract sample positive for *Aspergillus* spp., according to the Blot algorithm, adapted from Blot et al. [16]

	Immunosuppression (*n* = 17)^a^	No Immunosuppression (*n* = 18)
*Proven invasive pulmonary aspergillosis* (*n* *=* 1)	1 (6)	0 (0)
*Putative invasive pulmonary aspergillosis* (*n* = 16)	11 (65)	5 (28)^b^
1. Aspergillus-positive lower respiratory tract specimen culture	17	18
2. Compatible signs and symptoms		
Fever refractory to at least 3 d of appropriate antibiotic therapy	3	1
Recrudescent fever after a period of defervescence of at least 48 h while still on antibiotics and without other apparent cause	1	0
Pleuritic chest pain	1	0
Pleuritic rub	0	0
Dyspnea	0	0
Hemoptysis	1	0
Worsening respiratory insufficiency in spite of appropriate antibiotic therapy and ventilatory support	6	11
3. Abnormal medical imaging by portable chest X-ray or CT scan of the lungs	17	18
4a. Host risk factors	17	0
Neutropenia (absolute neutrophil count < 0.5 G/L) preceding or at the time of ICU admission	4	0
Underlying hematological or oncological malignancy treated with cytotoxic agents	5	0
Glucocorticoid treatment (prednisone equivalent >20 mg/d and >4 weeks)	1	0
Congenital or acquired immunodeficiency	7	0
4b. Semiquantitative *Aspergillus*-positive culture of BAL fluid (+ or ++), without bacterial growth together with a positive cytological smear showing branching hyphae	4	6
*Aspergillus respiratory tract colonization* (*n* = 18)	5 (29)	13 (72)^c^

^a^Hematological malignancies (*n* = 7, including lymphoma (*n* = 5), acute leukemia (*n* = 2), one of whom required allogeneic bone marrow transplant), solid organ transplant (*n* = 6), gastric cancer (*n* = 1), HIV infection (*n* = 1), neutropenia of unknown cause (*n* = 1) and connective tissue disease under corticosteroid treatment (*n* = 1)

^b^ *p* = 0.018 and ^c^ *p* = 0.015 (Fisher’s exact test) for comparison between immunosuppressed and non-immunosuppressed patients; continuous variables are shown as median (interquartile range 25–75); categorical variables are shown as *n* (%)

### Collection of data and definitions

Demographics and clinical characteristics upon ICU admission and during ICU stay were abstracted from the medical charts of all patients. Immunosuppression was defined by one of the following conditions: neutropenia (absolute neutrophil count <500 G/L) preceding or at the time of ICU admission, underlying hematological or oncological malignancy treated with cytotoxic agents, glucocorticoid treatment (prednisone equivalent >20 mg/day for more than 4 weeks), congenital (e.g., chronic granulomatous disease, hyper-IgE syndrome [17]) or acquired (e.g., AIDS [18]) immunodeficiency. Patient initial severity was assessed using the Simplified Acute Physiology Score II (SAPS II) [19] and Logistic Organ Dysfunction (LOD) [20] scores. ARDS was categorized as mild, moderate or severe according to the lowest PaO_2_/FiO_2_ ratio obtained within 48 h of ICU admission [12]. Shock was defined as need for vasopressor (epinephrine or norepinephrine) at a dose higher than 1 mg/h for more than 2 h. Outcome variables included the use of adjuvant therapies for ARDS (i.e., neuromuscular blocking agents, nitric oxide inhalation, prone positioning or venovenous extracorporeal membrane oxygenation), the need for renal replacement therapy or vasopressors, the administration of corticosteroids, the number of ventilator-free days at day 28, the duration of ICU stay, the incidence of ventilator-associated pneumonia and in-ICU mortality.

All chest CT scans performed in Aspergillus^+^ patients were reviewed by two pulmonologists (FS and NDP) blinded to the final *Aspergillus* classification and outcome. Elementary lesions including alveolar consolidation, lung nodules, ground-glass opacities, halo sign, cavitation and pleural effusion were recorded.

### Patient’s management

ARDS patients received mechanical ventilation using a standardized protective ventilation strategy [21, 22]. Tracheal suction was performed using a closed system. Other treatments including neuromuscular blocking agents [23], nitric oxide inhalation, prone positioning [24] and venovenous extracorporeal membrane oxygenation were administered depending on the severity of ARDS [25].

Antifungal therapy (voriconazole, caspofungin or liposomal amphotericin B) was administered at the discretion of the managing physician and not initiated on the sole basis of a positive GM in serum or in BAL fluid.

### Statistical analysis

Continuous variables are reported as median [25th–75th percentiles] or mean ± standard deviation (SD) and compared as appropriate. Categorical variables are reported as numbers and percentages [95 % confidence interval (95 % CI)] and compared as appropriate. There was no imputation for missing data, except for data missing from comorbidities, which were then considered as absent. Factors associated with ICU mortality were determined by univariable and multivariable backward logistic regression analyses. Independent variables with a *p* value <0.10 in univariable analysis were entered into the multivariable model, with backward elimination of variables displaying a *p* value greater than 0.05. Interactions between variables were assessed using the Mantel–Haenszel test. Analyses were conducted using the SPSS Base 21.0 statistical software package (SPSS Inc., Chicago, IL).

## Results

### Prevalence of *Aspergillus*^+^ respiratory tract samples during ARDS

**Fig. 1:**
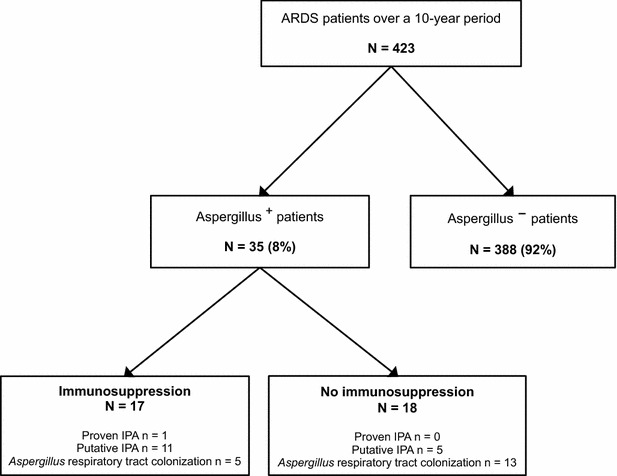
Flowchart of patients with the acute respiratory distress syndrome (ARDS) included in the study. Eight percent of patients (*n* = 35) had a respiratory tract culture positive for *Aspergillus* spp., including both immunosuppressed (*n* = 17) and non-immunosuppressed (*n* = 18) patients. The diagnostic probability of invasive pulmonary aspergillosis was assessed using the algorithm of Blot et al. [16]

Over the 10-year study period, 423 patients were admitted for ARDS, of whom 35 [8.3 %, 95 % CI (5.4–10.6)] had at least one respiratory tract sample positive for *Aspergillus* spp. (Aspergillus^+^ patients) (Fig. 1; Table 1).

Among 17 (49 %) immunocompromised Aspergillus^+^ patients, one had proven IPA, 11 had putative IPA, and 5 were categorized as having respiratory tract colonization. Conversely, among 18 (51 %) non-immunocompromised Aspergillus^+^ patients, 5 had putative IPA and 13 had colonization (Fig. 1; Table 2). The overall prevalence of proven/putative aspergillosis was 4.0 % [95 % CI (2.1–5.9)].

### Presentation of ARDS patients with *Aspergillus*-positive respiratory tract samples

Comorbidities did not differ between Aspergillus^+^ and Aspergillus^−^ patients except for more frequent immunosuppression in the former group (Table 1). The two groups did not differ regarding clinical presentation and severity of illness upon ICU admission, as assessed by SAPS II, LODS and ARDS severity. Regarding the main ARDS risk factors retrieved, infective pneumonia was significantly more frequent (while aspiration pneumonitis was less frequent) in Aspergillus^+^ patients than in others (Table 1).

Among the 35 patients of the Aspergillus^+^ group, 27 (77 %) had a GM measurement performed in both plasma and BAL fluid. Plasma GM measurements were not significantly different between patients with proven/putative IPA and those with *Aspergillus* spp. colonization (7/15, 47 % vs. 2/12, 17 %, *p* = 0.22). In contrast, when measured in BAL fluid, GM was more frequently positive in patients with proven/putative IPA than in those with *Aspergillus* colonization (8/15, 53 % vs. 0/12, 0 %, *p* = 0.003) (Table 3).

**Table 3:** Serum and bronchoalveolar lavage fluid galactomannan antigen according to the probability of invasive pulmonary aspergillosis (Blot et al. algorithm [16])

	All (*n* = 27)	Proven/putative aspergillosis (*n* = 15)	*Aspergillus* colonization (*n* = 12)	*p* value^a^
Positive serum galactomannan	9 (33)	7 (47)	2 (17)	0.22
Positive BAL fluid galactomannan	8 (30)	8 (53)	0 (0)	0.003

*BAL* bronchoalveolar lavage

^a^ *p* value comes from the Fisher exact test; an optical density (OD) ratio of 0.5 or greater for galactomannan antigen in serum and 1.0 for BAL fluid was considered positive

**Fig. 2:**
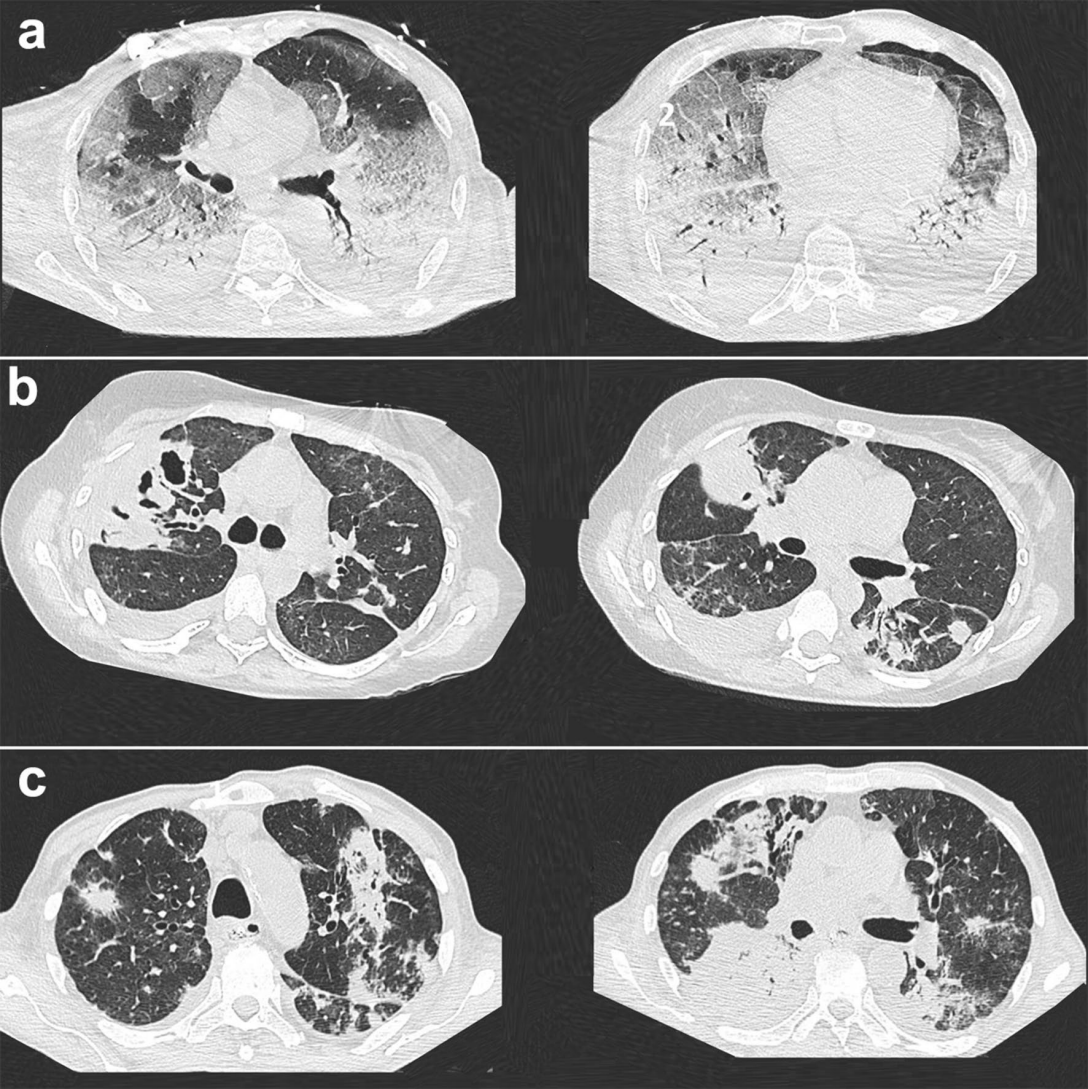
Chest CT scan images in patients with ARDS and one or more respiratory tract culture positive for *Aspergillus* spp., categorized as having putative invasive pulmonary aspergillosis (IPA) or *Aspergillus* colonization [16]. CT scan slices depicted **a** ARDS-typical bilateral basal consolidations, together with ground-glass opacities (*left panel*) and left anterior pneumothorax (*right panel*) in a patient categorized as having putative IPA; **b** *right upper* lobe cavitation (*left panel*), together with nodular lesions (*right panel*) in a patient with necrotizing group A *Streptococcus*, categorized as having *Aspergillus* respiratory tract colonization; and **c** nodular lesions with ground-glass opacities (*left panel*) and alveolar consolidations (*right panel*) in a patient categorized as having putative IPA

Chest CT scans were obtained in 60 % (*n* = 21/35) of patients of the Aspergillus^+^ group during ICU stay (Table 4; Fig. 2) and displayed no significant difference between patients categorized as having proven/putative aspergillosis (*n* = 13/21) and those with *Aspergillus* colonization (*n* = 8/21). Of note, while lung nodules were observed in 67 % of cases, other chest CT scan patterns suggestive of IPA, including lung cavitation and halo sign, were detected in only 14 % of cases. Alveolar consolidations, consistent with the underlying ARDS, were present in 90 % of cases.

**Table 4:** Chest CT scan patterns in patients (*n* = 21) categorized as having proven/putative invasive pulmonary aspergillosis or *Aspergillus* colonization, according to the Blot algorithm [16]

	All (*n* = 21)	Proven/putative aspergillosis (*n* = 13)	*Aspergillus* colonization (*n* = 8)	*p* value^a^
Pulmonary infiltrates	21 (100)	13 (100)	8 (100)	>0.99
Alveolar consolidation	19 (90)	11 (85)	8 (100)	0.11
Lung nodules	15 (71)	8 (61)	7 (87)	0.33
Ground-glass opacities	14 (67)	10 (77)	4 (50)	0.34
Cavitation	3 (14)	1 (8)	2 (25)	0.53
Halo sign	3 (14)	2 (15)	1 (12)	>0.99
Pleural effusion	12 (57)	8 (61)	4 (50)	0.67

^a^ *p* value comes from the Fisher exact test; categorical variables are shown as *n* (%)

### Management and outcome of ARDS patients with *Aspergillus*-positive respiratory tract samples

The median number of collected samples was 3 (2–7) per patient, and the median delay between ICU admission and the first respiratory tract sample positive for *Aspergillus* spp. was 3 days (1–11) (Table 5). There were no differences between Aspergillus^−^ and Aspergillus^+^ patients regarding duration of ICU stay, in-ICU mortality, number of ventilator-free days at day 28 and incidence of ventilator-acquired pneumonia and of shock. In contrast, the need for renal replacement therapy was almost twice as high in Aspergillus^+^ patients than in others (Table 5). Within the Aspergillus^+^ group, fifteen patients received an antifungal treatment during ICU stay (voriconazole, *n* = 12; liposomal amphotericin B, *n* = 3; caspofungin, *n* = 2; combination therapy, *n* = 3), including the sole patient with proven IPA, 10 over 16 patients with putative IPA and 4 over 18 patients with *Aspergillus* respiratory tract colonization.

**Table 5:** Management and outcomes of ARDS patients with (*Aspergillus* ^+^) or without (*Aspergillus* ^−^) one or more respiratory tract sample positive for *Aspergillus* spp.

	All (*n* = 423)	*Aspergillus* ^*−*^ (*n* = 388)	*Aspergillus* ^+^ (*n* = 35)	*p* value
Microbiological examinations				
Number of endobronchial samples	4.0 (2.0–7.0)	3.5 (2.0–7.0)	4.5 (2.7–9.2)	0.019
Including BAL	211 (48)	181 (45)	30 (86)	<0.0001
Duration of ICU stay (days)	12 (6–22)	12 (6–22)	14 (7–35)	0.14
Ventilator-free days at day 28 (days)	0 (0–17)	0 (0–22)	0 (0–16)	0.19
Ventilator-acquired pneumonia	146 (35)	135 (35)	11 (31)	0.85
Treatment				
Prone position	169 (40)	153 (40)	16 (46)	0.48
Nitric oxide inhalation	117 (28)	108 (28)	9 (26)	0.85
Paralyzing agents	380 (92)	348 (92)	32 (91)	>0.99
ECMO	21 (5)	18 (5)	3 (9)	0.40
Shock	350 (83)	321 (83)	29 (83)	>0.99
Renal replacement therapy	122 (29)	105 (27)	17 (49)	0.011
Corticosteroids				
“Stress-dose” steroids^a^	144 (34)	134 (34)	10 (29)	0.58
“High-dose” steroids^b^	96 (23)	84 (22)	12 (34)	0.094
In-ICU mortality	209 (50)	188 (48)	21 (60)	0.22

*ECMO* extracorporeal membrane oxygenation, *BAL* bronchoalveolar lavage

^a^Hydrocortisone 200 mg/day

^b^Prednisone equivalent >1 mg/kg/day; continuous variables are shown as median (interquartile range 25–75); categorical variables are shown as *n* (%)

The association between *Aspergillus* status, as categorized with the Blot et al. algorithm, and in-ICU mortality was assessed by logistic regression analysis. Both in univariable analysis [OR = 7.98 (1.80–35.36), *p* = 0.006)] and after adjusting for covariates significantly associated with ICU mortality, putative/proven IPA was strongly associated with in-ICU mortality [aOR = 9.58 (1.97–46.52), *p* = 0.005], while *Aspergillus* colonization was not [aOR = 0.64 (0.21–1.99), *p* = 0.44] (Table 6). Of note, within the putative/proven IPA subgroup (*n* = 17), 10/12 immunocompromised and 5/5 non-immunocompromised patients died in the ICU.

**Table 6:** Univariable and multivariable logistic regression analyses of factors associated with ICU mortality in ARDS patients

	*n*	Death *n* (%)	Univariable analysis		Multivariable analysis	
			OR (95 % CI)	*p*	aOR (95 % CI)	*p*
Age (years)	–	–	1.02 (1.01–1.03)	<0.0001	1.02 (1.00–1.03)	0.029
Year of inclusion	–	–	0.89 (0.82–0.95)	<0.001	–	–
Liver cirrhosis						
Yes	44	31 (70.5)	2.69 (1.37–5.31)	0.004	2.62 (1.24–5.54)	0.012
No	379	178 (47.0)	1		1	
Immunosuppression						
Yes	100	58 (58.0)	1.57 (1.00–2.47)	0.050	1.83 (1.08–3.11)	0.024
No	323	151 (46.7)	1		1	
PaO_2_/FiO_2_ ratio (mmHg)	–	–	0.99 (0.99–0.99)	<0.0001	0.99 (0.99–0.99)	<0.0001
SAPS II	–	–	1.03 (1.02–1.04)	<0.0001	1.02 (1.00–1.03)	0.018
LODS	–	–	1.19 (1.13–1.25)	<0.0001	1.12 (1.05–1.20)	<0.001
Antifungal treatment^a^						
Yes	17	12 (70.6)	2.55 (0.88–7.36)	0.084	–	–
No	406	197 (48.5)	1			
Blot et al. algorithm[16]						
No *Aspergillus* spp. colonization	388	188 (48.5)	1	–	1	–
*Aspergillus* spp. colonization	18	6 (33.3)	0.53 (0.20–1.45)	0.22	0.64 (0.21–1.99)	0.44
Putative or proven IPA	17	15 (88.2)	7.98 (1.80–35.36)	0.006	9.58 (1.97–46.52)	0.005

*IPA* invasive pulmonary aspergillosis

^a^As prescribed for a suspicion of invasive pulmonary aspergillosis; the Hosmer–Lemeshow goodness of fit test showed good calibration of the model (*p* = 0.28); the area under the curve of the model is 0.78 (0.73–0.82); OR (95 % CI), odds ratio (95 % confidence interval); aOR, adjusted odds ratio

## Discussion

We herein report *Aspergillus*-positive lower respiratory tract specimen culture in an 8 % prevalence of patients with ARDS, half of whom had putative or proven IPA. Immunosuppression and pneumonia were more frequent among patients having at least one positive sample for *Aspergillus*. Immunocompromised ARDS patients were more frequently categorized as having putative or proven IPA, while non-immunocompromised patients were more likely categorized as having *Aspergillus* respiratory tract colonization. Importantly, patients with one or more positive respiratory tract sample for *Aspergillus* had a worse outcome than others only when categorized as having putative/proven IPA according to the Blot algorithm.

The current series is, to the best of our knowledge, the largest one to focus on *Aspergillus*-positive respiratory tract samples in ARDS patients. The 8 % prevalence of patients having at least one positive sample for *Aspergillus,* in our population with ARDS, is significantly higher than the 1 % rate prospectively reported by Bassetti et al. in unselected mechanically ventilated patients, suggesting ARDS is a risk factor for *Aspergillus* respiratory colonization and/or infection [14]. Four percent of our ARDS patients (*n* = 17/423) were eventually classified as having proven or putative IPA, which is less than the 13 % prevalence of proven IPA that was previously reported in an autopsy study of 64 patients with ARDS [15], likely due to differences in case-mix and methods between this study and ours. Such figures are consistent with the fact that the Blot et al. algorithm was previously shown to have 61 % specificity and positive predictive value and 92 % sensitivity and negative predictive value, implying that its ability to exclude IPA might be better than in diagnosing it [16, 26]. Strikingly, the median delay between the first respiratory sample positive for *Aspergillus* spp. and mechanical ventilation initiation was 3 days, consistent with a previous study in mechanically ventilated non-ARDS patients [11], suggesting that respiratory tract colonization by *Aspergillus* spores had occurred prior to ARDS onset. The combination of ARDS-associated alveolar damage and associated local immune dysregulation [27], together with sepsis-induced immunosuppression [28], might, through alterations in innate immunity and antigen presentation processes [29], account for the development of IPA in previously colonized patients. Other previously described conditions at risk of IPA in critically ill non-immunosuppressed patients include COPD, present in only 11 % of our Aspergillus^+^ group, as compared to 31 % in a large series and, to a lesser extent, cirrhosis and corticosteroids, observed in less than 10 % of cases [6]. Surprisingly, however, corticosteroid administration was not associated with mortality in a recent series of mechanically ventilated patients with proven or putative Aspergillosis [6]. Although we found a trend toward more high-dose steroids administration in the Aspergillus^+^ group, their relationship with subsequent IPA and death could not be assessed in our study due to its limited statistical power.

The recent clinical algorithm proposed by Blot et al. for discriminating between ICU patients with *Aspergillus* respiratory tract colonization and those with IPA, allows for categorizing non-immunocompromised patients as having putative IPA, provided semiquantitative culture of BAL fluid is positive for *Aspergillus*, together with a positive cytological smear showing branching hyphae [16]. This criterion (4b) becomes indeed crucial in non-immunocompromised ARDS patients who all meet, by definition, the radiological criterion of the Blot algorithm (criterion 3), while both the relevance and reproducibility of several of the clinical criteria (e.g., dyspnea, pleuritic chest pain, pleuritic rub) can be questioned in critically ill mechanically ventilated patients. Nevertheless, and as expected, immunosuppression was strongly associated with proven/putative IPA in our series; however, it is noteworthy that non-immunocompromised patients accounted for one-third of patients classified as having probable infection, all of whom (*n* = 5/5) eventually died, suggesting putative IPA portends a dismal prognosis even in non-immunocompromised patients.

Although the purpose of our study was not to evaluate the performance value of GM antigen measurement, our results suggest that its detection is more efficient in BAL fluid than in plasma to discriminate between proven/putative IPA and *Aspergillus* colonization, in line with a previous prospective study conducted in non-ARDS critically ill patients [30]. In the context of ARDS patients with a positive culture for *Aspergillus,* a positive GM test in BAL fluid may be a helpful tool to reinforce the diagnostic suspicion of IPA and may thus incite clinicians to start antifungal therapy.

While the number of chest CT scans available in the current study was limited, our results suggest that, in the particular context of ARDS, its diagnostic yield to discriminate between putative aspergillosis and *Aspergillus* colonization is limited, most patients exhibiting non-specific findings such as alveolar consolidations.

In our series, the overall positivity of one or more respiratory sample for *Aspergillus* was not significantly associated with higher in-ICU mortality. Still, the risk of in-ICU mortality was significantly higher in ARDS patients with proven/putative IPA, as opposed to those with *Aspergillus* colonization, and as compared to those having no positive respiratory tract culture for *Aspergillus*, even after adjusting on significantly associated covariables. The benefit/risk ratio of antifungal therapy has not been assessed in ICU patients when categorized as having proven/putative IPA according to the recently proposed algorithm [16]. Our findings of a higher in-ICU mortality among a cohort of ARDS patients suggest that the initiation of such treatment should be considered in this specific subgroup, including non-immunocompromised patients, who also exhibited a strikingly high ICU mortality (*n* = 5/5 died). Of note, a previous observational study in critically ill COPD patients having putative IPA reported no improvement in ICU and long-term mortality in patients receiving antifungal treatment as compared to others, suggesting the severity of the underlying diseases was a key prognostic factor [7]. Strikingly, in the current series, six patients of the putative IPA subgroup (*n* = 16) did not receive an antifungal treatment, reflecting the fact that the criteria on which such treatment should be initiated in patients having *Aspergillus* spp.-positive respiratory tract samples are not standardized yet.

Our study has a number of limitations. First, due to its monocentric design, our results may not be applicable to other centers, thereby limiting their generalizability, since risk exposure to *Aspergillus*, prevalence of colonization and subsequent IPA may vary between centers. Moreover, the number and the type of respiratory tract samples performed were not standardized over the study period, potentially hampering the isolation of *Aspergillus* spp. in patients having had limited microbiological investigations. Second, this was a retrospective study with possible associated errors in data abstraction. However, due to the relatively low frequency of IPA, prospective studies in the specific subgroup of ARDS patients would be hardly feasible due to the low rate of *Aspergillus* colonization [8]. Third, our patients were admitted over a 10-year period, with inherently associated selection bias related to variations in coding habits between years. Moreover, during this relatively long time period, exposure to *Aspergillus* spores might have varied due to environmental factors. However, we found no association between the year of ICU admission and the risk of having one or more respiratory tract sample positive for *Aspergillus* spp. Fourth, several known prognostic factors for ARDS, including pulmonary artery pressure level or right ventricular dysfunction [31], were not available due to the retrospective nature of the study. Last, due to the limited number of patients having had a chest CT scan performed (*n* = 21/35), our study does not allow for drawing definite conclusions regarding the performance of chest CT scan in discriminating between putative aspergillosis and *Aspergillus* colonization in the context of ARDS.

## Conclusions

We report a prevalence of 8 % of *Aspergillus*-positive lower respiratory tract specimen culture and 4 % of proven or putative IPA during ARDS. Immunocompromised ARDS patients were more likely to be categorized as having a putative or proven IPA, while non-immunocompromised patients were more frequently classified as having *Aspergillus* respiratory tract colonization. Immunosuppression and pneumonia were associated with having at least one positive sample for *Aspergillus*. In this cohort of ARDS patients, having one or more positive sample for *Aspergillus* had no impact on outcome when classified as a mere respiratory tract colonization. In contrast, patients classified as having putative/proven IPA had a higher risk of in-ICU mortality, suggesting antifungal treatment should be assessed in this subgroup.

## Abbreviations

ARDS: acute respiratory distress syndrome
ICU: intensive care unit
IPA: invasive pulmonary aspergillosis
